# Vectorization of biomacromolecules into cells using extracellular vesicles with enhanced internalization induced by macropinocytosis

**DOI:** 10.1038/srep34937

**Published:** 2016-10-17

**Authors:** Ikuhiko Nakase, Kosuke Noguchi, Ikuo Fujii, Shiroh Futaki

**Affiliations:** 1 Nanoscience and Nanotechnology Research Center, Research Organization for the 21st Century, Osaka Prefecture University, Naka-ku, Sakai, Osaka 599-8570, Japan; 2 Graduate School of Science, Osaka Prefecture University, Naka-ku, Sakai, Osaka 599-8531, Japan; 3 Institute for Chemical Research, Kyoto University, Uji, Kyoto 611-0011, Japan

## Abstract

Extracellular vesicles (EVs, exosomes) are approximately 30- to 200-nm-long vesicles that have received increased attention due to their role in cell-to-cell communication. Although EVs are highly anticipated to be a next-generation intracellular delivery tool because of their pharmaceutical advantages, including non-immunogenicity, their cellular uptake efficacy is low because of the repulsion of EVs and negatively charged cell membranes and size limitations in endocytosis. Here, we demonstrate a methodology for achieving enhanced cellular EV uptake using arginine-rich cell-penetrating peptides (CPPs) to induce active macropinocytosis. The induction of macropinocytosis via a simple modification to the exosomal membrane using stearylated octaarginine, which is a representative CPP, significantly enhanced the cellular EV uptake efficacy. Consequently, effective EV-based intracellular delivery of an artificially encapsulated ribosome-inactivating protein, saporin, in EVs was attained.

Extracellular vesicles (EVs, exosomes) are cellular vesicles (30–200 nm in diameter) with a lipid bilayer that are constitutively secreted from most cells. EVs are found in abundance in bodily fluids, such as blood, saliva, urine, and breast milk^1^,^2^,^3^. In cell-to-cell communication, EVs transport genetic materials (e.g., microRNAs) and enzymes to other cells, resulting in signal transduction for cell regulation and the modulation of the immune response^1^,^2^,^3^. Secreted EVs can transport biologically functional molecules to other cells via endocytosis, such as tetraspanin membrane proteins (CD9, CD63, CD81, CD82), proteins involved in multivesicular body biogenesis (Alix, TSG101), heat-shock proteins (Hsp70, Hsp90), other bioactive proteins (GTPases, annexins, flotillin), and raft-associated lipids (e.g., cholesterol, ceramide, sphingolipids, phosphoglycerides, phospholipases) encapsulated in EVs^4^,^5^. EVs have been examined as intracellular delivery carriers of therapeutic genes for cellular regulation^3^,^6^,^7^,^8^. However, to achieve the effective intracellular delivery of EV contents, the cellular uptake efficacy of EVs must increase, as many EVs are present in bodily fluids, leading to competition for cellular uptake. Moreover, the negative charge of EV membranes prevents them from accumulating on negatively charged cellular membranes^9^,^10^.

Endocytosis has been shown to be a major route for cellular EV uptake^11^,^12^,^13^,^14^. Clathrin-mediated endocytosis has a size limitation (approximately 120 nm) for cellular uptake from outside of the cells^15^, and this route of cellular uptake is considered inefficient for the cellular uptake of EVs (which are approximately 200 nm in size). However, our research group recently reported that active induction of macropinocytosis (accompanied by actin reorganization, ruffling of the plasma membrane, and engulfment of large volumes of extracellular fluid)^16^,^17^ by cancer-related receptors (e.g., epidermal growth factor receptor) and the expression of oncogenic K-Ras significantly enhances cellular EV uptake^9^. Combined treatment of EVs with ligands for macropinocytosis induction (e.g., epidermal growth factor) increases the cellular EV uptake efficacy; however, this experimental technique is considered ineffective in *in vivo* treatment due to the dispersal of EVs in bodily fluid. Thus, the artificial induction of macropinocytosis stimulated by the functionalized EV itself is strongly considered very useful for the EV-based intracellular delivery of therapeutic molecules.

Given the importance of the artificial induction of macropinocytosis for the development of EV-based intracellular delivery systems, in this study, we demonstrate that modification of arginine-rich cell-penetrating peptides (CPPs) on EV membranes results in the effective induction of macropinocytosis and cellular EV uptake (Fig. 1). Arginine-rich CPPs, including human immunodeficiency virus type 1 (HIV-1) Tat (48–60) peptide and oligoarginine peptides, have been reported to be promising carriers for the intracellular delivery of various bioactive molecules, such as proteins, peptides, and nucleic acids^18^,^19^. Macropinocytosis has also been shown to be an important pathway for the physiological cellular uptake of arginine-rich CPPs^20^,^21^,^22^,^23^,^24^. Recently, our research group also found that the octaarginine peptide, which is a representative arginine-rich CPP, induces the clustering of the syndecan-4 proteoglycan on plasma membranes, resulting in the binding of PKCα to the V domain of the proteoglycan in the cytosol and the activation of PKCα^25^. The induction of proteoglycan clustering and PKCα activation results in the induction of macropinocytosis and the effective cellular uptake of the peptide^25^. In this report, we propose a simple and effective technique for enhancing the cellular uptake of EVs using arginine-rich CPPs. The modification of arginine-rich CPPs highly enhances the cellular uptake of EVs via the active induction of macropinocytosis without any cytotoxicity. We also achieved the efficient cytosolic delivery of a ribosome-inactivating protein, saporin, using arginine-rich CPP-modified EVs, leading to the efficient induction of cytotoxicity in targeted cells.

## Results and Discussion

### Effect of stearyl-r8 modification on the cellular uptake of EVs

**Figure 1:**
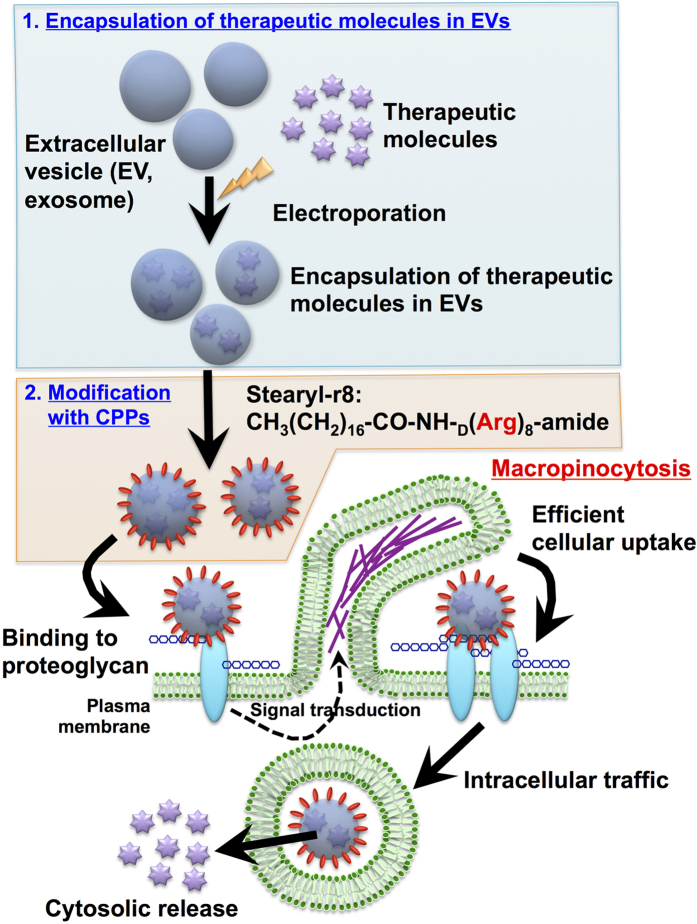
Schematic representation of the intracellular delivery of EVs with the modification of arginine-rich cell-penetrating peptide for the active induction of macropinocytosis. Objective therapeutic molecules are encapsulated in EVs by electroporation. EVs are then modified with stearyl-r8 peptide on EV membranes, resulting in the active induction of macropinocytosis and effective cellular uptake.

**Figure 2:**
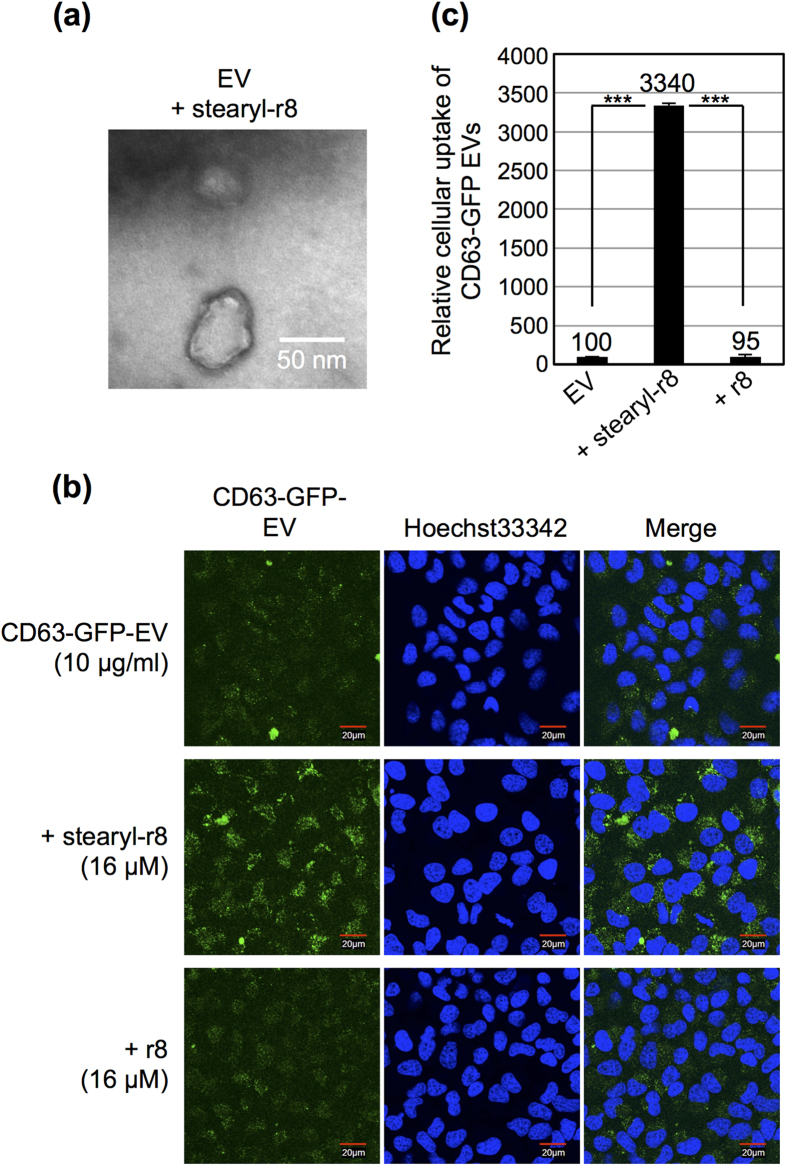
Increased cellular uptake of EVs by modification with stearyl-r8 peptide. (**a**) TEM observation of CD63-GFP EVs (10 μg/ml) modified with stearyl-r8 (16 μM). (**b**) Confocal microscopic observation of HeLa cells treated with CD63-GFP EVs (10 μg/ml) modified with stearyl-r8 (16 μM) or r8 (16 μM) for 24 h at 37 °C (blue: Hoechst 33342; green: CD63-GFP EVs). (**c**) Relative cellular uptake of CD63-GFP EVs (10 μg/ml) modified with stearyl-r8 (16 μM) or r8 (16 μM) analyzed using a flow cytometer under the same experimental conditions as in (**b**). The data are expressed as the average (±SD) of three experiments. ^***^p < 0.001.

In our system, EV membranes were modified with r8, which is a representative arginine-rich CPP^18^,^19^, by simple mixing with stearyl-r8, where the stearyl moiety served as an anchoring unit to membranes (Fig. 1). Tetraspanin CD63 is a marker membrane protein of the EV, and green fluorescent protein (GFP)-fused CD63 stably expressing HeLa cells (CD63-GFP-HeLa) (Supplementary Fig. 1) were prepared in these experiments. Secreted CD63-GFP EVs were isolated from the cell culture medium via ultracentrifugation methods^26^, and the expression of the EV marker protein CD63 was detected using western blot analysis (Supplementary Fig. 1c). After the EVs were mixed with the peptide, the removal of excess stearyl-r8 peptide, which was not modified on EV membranes, was accomplished by washing and filtration using centrifugal filters. The zeta-potential of the stearyl-r8-modified EV was shown to be 5.1 mV, and the average diameter of the EVs was 196.9 ± 46.7 nm, as determined using the zeta-potential and a particle size analyzer (Supplementary Fig. 1e). Conversely, the average diameter of the original isolated EVs without modification of the stearyl-r8 peptide was 116.4 ± 49.2 nm (Supplementary Fig. 1d), and the zeta-potential of isolated EVs was −12.5 mV, which is similar to previously reported values^9^,^10^, suggesting an increase in the charge of the EV membrane by the modification of the stearyl-r8 peptide. Transmission electron microscopy (TEM) observations of the stearyl-r8-modified EV showed vesicular structures similar to the original isolated EVs without modification of the stearyl-r8 peptide (Fig. 2a and Supplementary Fig. 1b).

Next, the cellular uptake efficacy of the stearyl-r8-modified EV was evaluated using confocal microscopy and flow cytometry. Marked induction of macropinocytosis, which facilitated the cellular uptake of EVs into cells, was attained by surface modification with stearyl-r8 peptide, as was also observed for liposomes modified with octaarginine^27^,^28^,^29^. Figure 2b shows a confocal microscopic image of HeLa cells (derived from human cervix adenocarcinoma) treated with CD63-GFP-EVs (10 μg/ml) in 10% serum-containing cell culture medium for 24 h at 37 °C. When the cells were treated with CD63-GFP-EVs without modification of stearyl-r8 peptide, very low cellular uptake efficacy of the EVs was observed (Fig. 2b). However, in the case of the modification of stearyl-r8 (16 μM) on EV membranes, comparatively highly fluorescent signals of CD63-GFP-EVs taken up by the cells were observed (Fig. 2b). Flow cytometry analysis also showed that the cellular EV uptake was 33-fold higher due to the modification of stearyl-r8 (Fig. 2c). When the cells were treated with CD63-GFP-EVs premixed with r8 peptide without the stearyl moiety, the cellular EV uptake efficacy was similar to that of the original EV (Fig. 2b,c), suggesting that the stearyl moiety is essential for the modification of the r8 peptide on EV membranes. The cellular uptake efficacy of EVs modified with stearyl-r8 peptide is dependent on the peptide concentration (Supplementary Fig. 2). A peptide concentration of 16 μM for the EV membrane modification (10 μg/ml EV) resulted in effective cellular uptake efficacy (Supplementary Fig. 2). However, in the case of a low concentration of stearyl-r8 (1.6 μM), the cellular EV uptake efficacy was only slightly affected (Supplementary Fig. 2). In addition, fluorescently labeled peptide (stearyl-r8-GC(Alexa488)) was used to confirm the binding of the stearyl-r8 peptide to EV membranes using a spectrofluorometer, and this method resulted in the binding of stearyl-r8-GC(Alexa488) (221 nM) on 10 μg/ml of EVs. However, in the case of r8-GC(Alexa488) without a stearyl moiety, this method resulted in less binding of r8-GC(Alexa488) (10 nM) on 10 μg/ml of EVs. We also confirmed the internalization of EVs by other cells, such as A431 cells (derived from human epidermoid carcinoma) and CHO-K1 cells (derived from Chinese hamster ovary) (Supplementary Fig. 4). The cellular uptake of CD63-GFP-EVs (without modification of stearyl-r8) into A431 cells was shown to have very low efficacy (23.4-fold and 17.8-fold lower than that of CHO-K1 and HeLa cells, respectively) in the experimental condition of CD63-GFP-EV (10 μg/ml) uptake in 10% serum-containing cell culture medium for 24 h at 37 °C. However, the modification of stearyl-r8 (16 μM) on the EV membrane had a significant effect on cellular EV uptake in A431 cells (Supplementary Fig. 4). The cell viability of the treatment with stearyl-r8-modified EVs was tested using the WST-1 assay, and almost no cytotoxicity was observed (Supplementary Figs 3 and 5).

### Active induction of macropinocytosis by stearyl-r8-modified EVs

**Figure 3:**
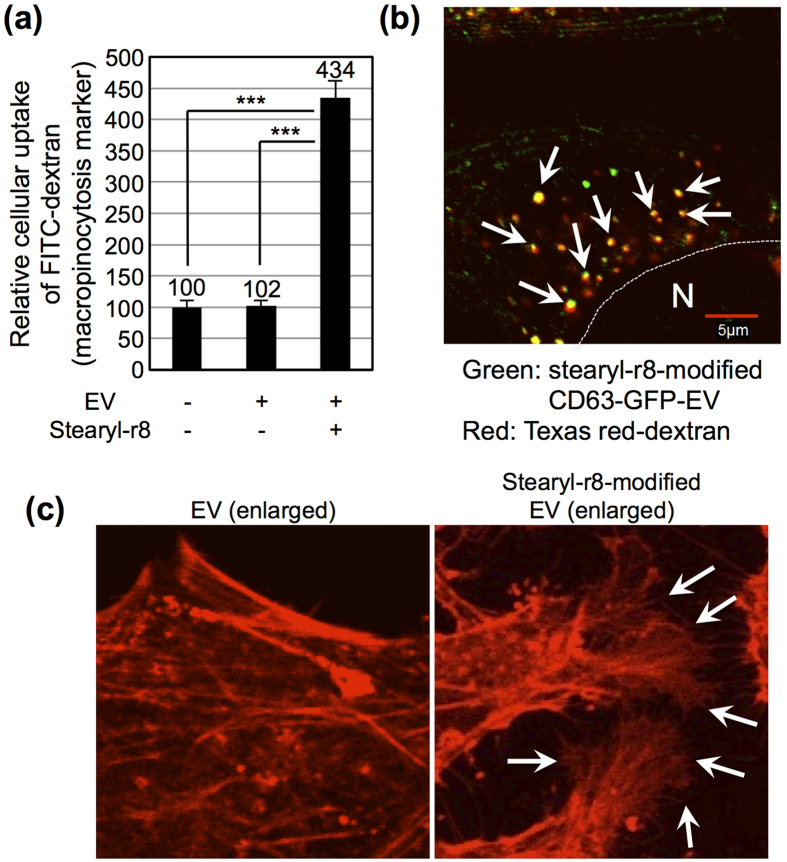
Active induction of macropinocytosis by the modification of stearyl-r8 on the EV membrane. (**a**) Relative cellular uptake of the macropinocytosis marker FITC-dextran in the presence or absence of EVs (10 μg/ml) with or without the modification of stearyl-r8 (16 μM) for 24 h at 37 °C analyzed using a flow cytometer. The data are expressed as the average (±SD) of three experiments. ^***^p < 0.001. (**b**) Confocal microscopic image of HeLa cells treated with CD63-GFP EVs (10 μg/ml) modified with stearyl-r8 (16 μM) and the macropinocytosis marker Texas red-dextran for 24 h at 37 °C (green: CD63-GFP EVs; red: Texas red-dextran). “N” shows the nucleus. The arrows show the representative colocalization of EV and dextran. (**c**) Confocal microscopic image of HeLa cells treated with CD63-GFP EVs (10 μg/ml) modified with stearyl-r8 (16 μM) or EV without the peptide modification for 20 min at 37 °C. Cellular staining with rhodamine-phalloidin was performed to visualize F-actin prior to observation. The original images are shown in Supplementary Fig. 9. The arrows show representative lamellipodia formations.

To examine the cellular uptake mechanisms of the stearyl-r8-modified EV, we tested the induction of macropinocytosis by the treatment of the EV on cells. First, we tested cellular EV uptake under low temperature (4 °C), which is an experimental condition for the prevention of endocytosis^21^. Supplementary Fig. 6a shows the flow cytometer analysis of HeLa cells treated with stearyl-r8-modified CD63-GFP-EV-containing cell culture medium for 2 h at 37 °C or 4 °C. In the case of the 4 °C treatment, the cellular uptake efficacy of stearyl-r8-modified CD63-GFP-EV was greatly reduced compared with the 37 °C treatment, which suggested that energy-dependent endocytosis is important for the cellular uptake of the EV. Next, we tested the macropinocytosis induction of stearyl-r8-modified EV analyzed using FITC-dextran (molecular weight: 70,000), which is a marker of macropinocytotic cellular uptake^20^,^30^. The activation of the epidermal growth factor receptor by epidermal growth factor (EGF) induces macropinocytosis^9^, and the EGF treatment on HeLa cells increased the cellular uptake of FITC-dextran (Supplementary Fig. 7). Figure 3a shows the results of a flow cytometer analysis of HeLa cells treated with FITC-dextran in the presence or absence of stearyl-r8-modified EVs. Co-treatment of FITC-dextran with stearyl-r8-modified EVs resulted in the enhanced cellular uptake of FITC-dextran (Fig. 3a). However, EVs without modification of the stearyl-r8 peptide did not increase the cellular uptake of FITC-dextran (Fig. 3a), which suggested that the modification of the stearyl-r8 peptide on EV membranes effectively enhanced the cellular uptake route of macropinocytosis. Confocal microscopic observations also showed the colocalization of Texas red-dextran and stearyl-r8-modified CD63-GFP-EV inside cells after their cellular uptake (Fig. 3b), which suggested cellular EV uptake via the macropinocytosis route. 5-(N-ethyl-N-isopropyl) amiloride^16^,^21^ (EIPA) is a representative macropinocytosis inhibitor, and the cellular uptake of the macropinocytosis marker FITC-dextran (molecular weight: 70,000) was decreased under the cellular treatment with EIPA (Supplementary Fig. S6b). The cellular uptake of stearyl-r8-modified CD63-GFP-EVs was significantly decreased by treatment with EIPA (Supplementary Fig. 6c). However, the EIPA treatment did not affect the cellular uptake of CD63-GFP-EVs without the modification of stearyl-r8 on the EV membranes (Supplementary Fig. S6d). We recently reported that the enhanced clustering of syndecan-4, a proteoglycan, by arginine-rich CPPs on plasma membranes is important for the binding of PKCα to the syndecan-4 V-domain inside cells and inducing macropinocytosis^25^. Thus, in this study, we also confirmed whether the clustering of syndecan-4 on the plasma membrane and stearyl-r8-modified EVs induced syndecan-4 clustering (Supplementary Fig. 8), similar to that observed using arginine-rich CPPs, as previously reported^25^. We also found that lamellipodia formation and membrane ruffling by actin organization could be observed when the cells were treated with stearyl-r8-modified EVs (Fig. 3c and Supplementary Fig. 9). However, EVs without peptide modification could not induce lamellipodia formation (Fig. 3c and Supplementary Fig. 9), which suggested that the modification of stearyl-r8 on the EV membrane is important for the active induction of membrane ruffling and macropinocytosis.

### Enhanced cellular uptake of bioactive protein-encapsulated EVs modified by stearyl-r8

Next, we examined the cellular uptake of cargo molecules artificially encapsulated in EVs. First, we prepared FITC-dextran (molecular weight: 70,000)-encapsulated EVs via electroporation^10^. Supplementary Fig. 10a shows confocal microscopic images of HeLa cells treated with FITC-dextran-encapsulated EVs for 24 h at 37 °C. The modification of the stearyl-r8 peptide on EV membranes enhanced their cellular uptake even at lower protein concentrations of EVs (1 μg/ml) than those employed in Fig. 2b. Under the same experimental conditions, the fluorescence intensity in the cells was analyzed using a flow cytometer (Supplementary Fig. 10b), and increased fluorescence intensity was detected from the FITC-dextran-encapsulated EVs modified with stearyl-r8 efficiently taken up by the cells (Supplementary Fig. 10b).

**Figure 4:**
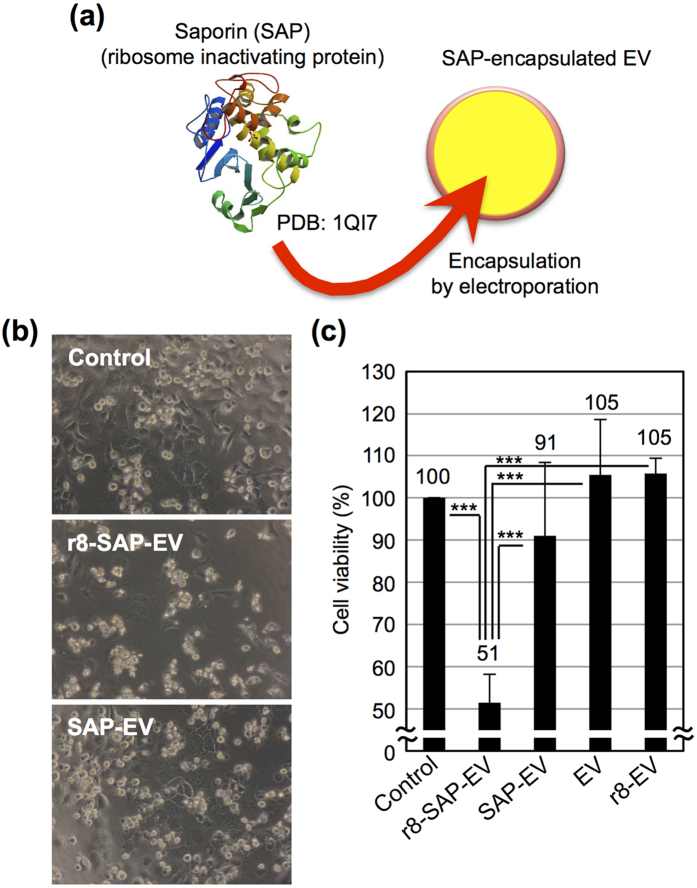
Enhanced biological activity of saporin encapsulated in EVs by the modification of stearyl-r8. (**a**) Schematic representation of the encapsulation of saporin (SAP) (PDB (Protein Data Bank) accession number: 1QI7)^32^ in EVs via electroporation. (**b**) Microscopic images of HeLa cells treated with saporin-encapsulated EVs (10 μg/ml) with or without the modification of stearyl-r8 (16 μM) (r8-SAP-EV and SAP-EV, respectively) for 72 h at 37 °C. The concentration of saporin encapsulated in 10 μg/ml EVs was estimated to be approximately 43.5 ng/ml using the FITC-labeled saporin. (**c**) Cell viability was analyzed using the WST-1 assay under the same experimental conditions as in (**b**). The data are expressed as the average (±SD) of four experiments. ^***^p < 0.001.

In addition, we encapsulated the ribosome-inactivating protein saporin^31^,^32^, which functions as an anti-cancer drug, in EVs via electroporation using an experimental method similar to that used to encapsulate FITC-dextran in EVs (Fig. 4a). HeLa cells were treated with saporin-encapsulated EVs for 72 h at 37 °C prior to microscopic observation and WST-1 assay (Fig. 4b,c). With the modification of stearyl-r8 on EV membranes, the cytotoxicity of encapsulated saporin in EVs was significantly enhanced (Fig. 4b,c). However, saporin-encapsulated EVs without the modification of stearyl-r8 on EV membranes showed low cytotoxicity (Fig. 4b,c), which suggested that the enhanced cellular uptake and cytosolic release of saporin originally encapsulated in EVs was effectively achieved in the cytosol by the modification of stearyl-r8 on EV membranes.

## Conclusion

In this study, we successfully developed an EV-based intracellular delivery system via the effective induction of macropinocytosis by the modification of the arginine-rich cell-penetrating peptide on EV membranes. The macropinocytosis route can take up large molecules from outside cells (>1 μm)^15^, and this route is considered well-suited for cellular EV uptake due to its vesicular size^9^. Our simple method using stearyl-r8 peptide to anchor the hydrophobic moiety on EV membranes and to decorate the r8 peptide on objective EVs to induce active macropinocytosis is very useful for the effective intracellular delivery of biomacromolecules in EVs into targeted cells without any covalent conjugation to amino acid side chains of EV membrane proteins. Thus, this technique promisingly maintains the functionality of EV membrane proteins during their cellular uptake despite the modification of stearyl-r8 on the EV membranes. Our study is the first to apply this modification system to biologically generated cellular vesicles, EVs. Our results should provide fundamental knowledge to further establish EV-based intracellular delivery systems, and our findings contribute to not only the development of EV-based intracellular delivery but also our understanding of EV functionality.

## Methods

### Peptide synthesis

All peptides were chemically synthesized via 9-fluorenylmethyloxycarbonyl (Fmoc) solid-phase peptide synthesis on a Rink amide resin with a coupling system using 1-hydroxybenzotriazole (HOBt)/2-(1H-benzotriazole-1-yl)-1,1,3,3-tetramethyluronium hexafluorophosphate (HBTU) (Peptide Institute, Osaka, Japan)/N,N-diisopropylethylamine (DIEA) as previously described^23^,^33^. The Fmoc-amino acid derivatives and the Rink amide resin were purchased from the Peptide Institute (Osaka, Japan) and Shimadzu Biotech (Kyoto, Japan), respectively. For the preparation of the stearylated peptide, the N-terminus of the peptide resin was reacted with the stearyl acid with diisopropylcarbodiimide in the presence of HOBt as coupling agents as previously reported^33^. The deprotection of the protected peptide and cleavage from the resin were performed via treatment with a trifluoroacetic acid (TFA)/ethanedithiol (EDT) mixture (95:5) for 3 h at 20 °C, followed by purification with reverse-phase high-performance liquid chromatography (HPLC). Based on the analytical HPLC, the purity of each peptide was estimated to be >97%. The structures of the synthesized peptides were confirmed using matrix-assisted laser desorption ionization time-of-flight mass spectrometry (MALDI-TOFMS) (Microflex, Bruker, Billerica, MA, USA).

Stearyl-r8 (CH_3_(CH_2_)_16_-CO-NH-_D_(Arg)_8_-amide): MALDI-TOFMS: 1533.6 [calculated for (M + H)^+^: 1534.0]. Retention time in HPLC: 21.2 min (column: Cosmosil 5C18-AR-II (4.6 × 150 mm); gradient: 5–95% B in A (A = H_2_O containing 0.1% CF_3_COOH, B = CH_3_CN containing 0.1% CF_3_COOH) over 30 min; flow: 1 ml/min; detection: 220 nm). Yield from the starting resin: 28%.

r8 (NH_2_-_D_(Arg)_8_-amide): MALDI-TOFMS: 1267.1 [calculated for (M + H)^+^: 1267.5]. Retention time in HPLC: 13.5 min (column: Cosmosil 5C18-AR-II (10 × 250 mm); gradient: 5–80% B in A (A = H_2_O containing 0.1% CF_3_COOH, B = CH_3_CN containing 0.1% CF_3_COOH) over 30 min; flow: 1 ml/min; detection: 220 nm). Yield from the starting resin: 10.2%.

### Fluorescently labeled peptides

For preparation of the fluorescently labeled peptides, the peptide was designed to have a glycyl cysteine amide at the C-terminus. The deprotection of the protecting group and detachment of the peptide from the resin were performed using TFA–EDT (95:5) at 20 °C for 3 h, and the product was purified via HPLC purification. Fluorescent labeling with purified peptides was performed by treatment with 1.5 equivalents of Alexa Fluor 488 (Alexa488) C5 maleimide sodium salt (Invitrogen, Eugene, OR, USA) in a dimethyl formamide/methanol mixture (1:1) for 1.5 h at room temperature followed by HPLC purification, as previously reported^23^.

Stearyl-r8-GC (CH_3_(CH_2_)_16_-CO-NH-_D_(Arg)_8_-Gly-Cys-amide): MALDI-TOFMS: 1693.4 [calculated for (M + H)^+^: 1693.3]. Retention time in HPLC: 19.2 min (column: Cosmosil 5C18-AR-II (4.6 × 150 mm); gradient: 5–95% B in A (A = H_2_O containing 0.1% CF_3_COOH, B = CH_3_CN containing 0.1% CF_3_COOH) over 30 min; flow: 1 ml/min; detection: 220 nm). Yield from the starting resin: 0.4%.

r8-GC (NH_2_-_D_(Arg)_8_-Gly-Cys-amide): MALDI-TOFMS: 1426.8 [calculated for (M + H)^+^: 1426.9]. Retention time in HPLC: 8.4 min (column: Cosmosil 5C18-AR-II (4.6 × 150 mm); gradient: 5–95% B in A (A = H_2_O containing 0.1% CF_3_COOH, B = CH_3_CN containing 0.1% CF_3_COOH) over 30 min; flow: 1 ml/min; detection: 220 nm). Yield from the starting resin: 20.8%.

Stearyl-r8-GC(Alexa488) (CH_3_(CH_2_)_16_-CO-NH-_D_(Arg)_8_-Gly-Cys(Alexa488)-amide): MALDI-TOFMS: 2391.5 [calculated for (M + H)^+^: 2391.0]. Retention time in HPLC: 19.8 min (column: Cosmosil 5C18-AR-II (4.6 × 150 mm); gradient: 5–95% B in A (A = H_2_O containing 0.1% CF_3_COOH, B = CH_3_CN containing 0.1% CF_3_COOH) over 30 min; flow: 1 ml/min; detection: 220 nm). Yield from the starting purified stearyl-r8-GC peptide: 0.3%.

r8-GC(Alexa488) (NH_2_-_D_(Arg)_8_-Gly-Cys(Alexa488)-amide): MALDI-TOFMS: 2125.5 [calculated for (M + H)^+^: 2124.5]. Retention time in HPLC: 10.5 min (column: Cosmosil 5C18-AR-II (4.6 × 150 mm); gradient: 5–95% B in A (A = H_2_O containing 0.1% CF_3_COOH, B = CH_3_CN containing 0.1% CF_3_COOH) over 30 min; flow: 1 ml/min; detection: 220 nm). Yield from the starting purified r8-GC peptide: 13.5%.

### Cell cultures

HeLa (human cervical cancer-derived) cells were purchased from the Riken BRC Cell Bank (Ibaraki, Japan). The human epidermoid carcinoma-derived A431 cells and Chinese hamster ovary (CHO)-K1 cells were purchased from the American Type Culture Collection (Manassas, VA, USA). Each cell was cultured in α-MEM (Gibco, Life Technologies Corporation, Grand Island, NY, USA) (HeLa cells), minimum essential medium (MEM) (Gibco, Life Technologies Corporation) (A431 cells), and F-12 nutrient mixture (Ham’s F-12) (CHO-K1) (Gibco, Life Technologies Corporation) containing 10% heat-inactivated FBS (Gibco, Life Technologies Corporation). Each cell was grown on 100-mm dishes and incubated at 37 °C under 5% CO_2_.

### Preparation of HeLa cells stably expressing green fluorescent protein (GFP)-fused CD63

CD63 is a membrane marker tetraspanin protein of EVs, and we prepared HeLa cells stably expressing GFP-fused CD63 to secrete CD63-GFP-containing EVs (CD63-GFP-EVs) as previously reported^9^,^10^. HeLa cells (1 × 10^5^ cells) were plated on a 24-well microplate (Iwaki, Tokyo, Japan) and incubated for 1 day. They were transfected with CD63-GFP plasmid (pCT-CD63-GFP, pCMV, Cyto-Tracer, System Biosciences, Mountain View, CA) (800 ng) complexed with Lipofectamine LTX reagent (2 μl) and PLUS reagent (1 μl) (Invitrogen, Life Technologies Corporation) in α-MEM containing 10% FBS (200 μl). The cells were also treated with puromycin (3 μg/ml) (LKT Laboratories, St. Paul, MN) for the antibiotic selection of HeLa cells stably expressing CD63-GFP (CD63-GFP-HeLa).

### Isolation of EVs

CD63-GFP-HeLa cells or HeLa cells (without expression of CD63-GFP) (each 3 × 10^6^ cells) were seeded onto 100-mm dishes in α-MEM containing 10% exosome-free FBS (EXO-FBS, ATLAS biological, Fort Collins, CO, USA) for 3 days. The cell culture medium was collected, and the secreted EVs were isolated using ultracentrifugation as previously reported^26^. The collected cell culture medium was centrifuged (300 × *g*) for 10 min at 4 °C. The supernatant was centrifuged (2,000 × *g*) for 10 min at 4 °C and again centrifuged (10,000 × *g*) for 30 min at 4 °C to remove cell debris. The supernatant was then centrifuged (100,000 × *g*) for 70 min at 4 °C (Himac CP65β, Hitachi, Tokyo, Japan) in duplicate, and the pellet was collected in PBS. The concentrations of isolated EVs were described in terms of their protein concentrations, which were determined using a Pierce BCA protein assay kit (Thermo Fisher Scientific Inc., Rockford, IL, USA).

### Western blotting analysis

Isolated EVs were added to lysis buffer (62.5 mM Tris-HCl (pH = 6.8), 2% SDS, 10% glycerol, 0.002% bromophenol blue, 5% 2-mercaptoethanol). The boiled samples were separated via 10% SDS-PAGE, transferred onto polyvinylidene fluoride (PVDF) membranes (GE Healthcare, Pittsburgh, PA, USA), and treated with anti-CD63 antibody (TS63, Abcam, Cambridge, UK). A secondary antibody labeled with horseradish peroxidase (anti-rabbit IgG HRP-linked whole antibody donkey, GE Healthcare) was used, and immunoreactive species were detected using the Enhanced Chemiluminescence (ECL) Plus Western Blotting Detection System (GE Healthcare) with the Amersham Imager 600 (GE Healthcare). Immunoreactive species were detected at a position of approximately 50 kDa in SDS-PAGE analysis using the anti-CD63 antibody.

### Modification of EVs with stearyl-r8 peptide

Synthesized stearyl-r8 peptide (final 40 μM) diluted with phosphate-buffered saline (PBS) was added to a solution of EVs (100 μg) in PBS (total 800 μl) and incubated for 1 h at 37 °C. Removal of unattached stearyl-r8 peptide was accomplished by washing with PBS and filtration using Amicon Ultra centrifugal filters (100K device, Merck Millipore). The attachment of stearyl-r8-GC(Alexa488) and r8-GC(Alexa488) to EVs was confirmed using a spectrofluorometer (FP-6200, JASCO, Tokyo, Japan).

### Confocal microscopy

Cells (2 × 10^5^ cells, 2 ml) were plated onto a 35-mm glass dish (Iwaki, Tokyo, Japan) and incubated in cell culture medium (HeLa cells, α-MEM; A431 cells, MEM; CHO-K1 cells, Ham’s F-12) containing 10% FBS for 24 h at 37 °C under 5% CO_2_. After complete adhesion, the cells were washed with cell culture medium containing 10% FBS and treated with each EV sample (100 μl/well). The cells were stained with Hoechst 33342 dye (Invitrogen; 5 μg/ml) for 15 min at 37 °C prior to cell washing. The cells were then washed with fresh cell culture medium and analyzed using a FV1200 confocal laser scanning microscope (Olympus, Tokyo, Japan) equipped with a 40× or 60× objective without cell fixation. For the detection of macropinocytosis uptake, co-treatment with Texas red-dextran (molecular weight: 70,000, 0.5 mg/ml; Molecular Probes, Eugene, OR, USA) with EV samples was performed.

For the detection of syndecan-4 clustering, the cells were treated with EV (without CD63-GFP expression, 10 μg/ml) modified with or without stearyl-r8 (16 μM) for 1 h at 37 °C. After the removal of the medium, the cells were fixed with 4% paraformaldehyde at room temperature for 30 min and washed with PBS. The cells were then treated with 0.1% Triton X-100 (100 μl/well in PBS) at room temperature for 5 min and again washed with PBS. The expression of syndecan-4 was visualized by treatment with syndecan-4 antibody (sc-12766, Santa Cruz Biotechnology, Santa Cruz, CA), and secondary antibody labeled with fluorescence (Alexa Fluor 488 goat anti-mouse IgG (H + L), Invitrogen) was then used (each 30 min at room temperature) prior to analysis using a FV1200 confocal laser scanning microscope (Olympus).

### Flow cytometry

Cells (4.7 × 10^4^ cells, 1 ml) were plated onto a 24-well microplate (Iwaki) and incubated in cell culture medium (HeLa cells, α-MEM; A431 cells, MEM; CHO-K1 cells, Ham’s F-12) containing 10% FBS for 24 h at 37 °C under 5% CO_2_. After complete adhesion, the cells were washed with cell culture medium containing 10% FBS and treated with each EV sample or macropinocytosis marker, FITC-labeled dextran (molecular weight: 70,000, 0.5 mg/ml) (Sigma-Aldrich) (600 μl/well) prior to washing with 0.5 mg/ml heparin in PBS (triple washing, 200 μl). The cells were then treated with 0.01% trypsin at 37 °C for 10 min prior to the addition of PBS (200 μl) and then centrifuged at 1,500 rpm (200 × *g*) for 5 min at 4 °C. After the removal of the supernatant, the cells were washed with PBS (400 μl) and centrifuged at 1,500 rpm for 3 min at 4 °C. This washing cycle was repeated, and the cells were suspended in PBS (400 μl) and subjected to fluorescence analysis with a Guava easyCyte (Merck Millipore, Billerica, MA, USA) flow cytometer using 488-nm laser excitation and a 525-nm emission filter. Live cells (10,000 cells/sample) for the detection of cellular fluorescence intensity were quantified based on forward-scattering and side-scattering analyses. For induction of macropinocytosis in Supplementary Figs 6b and 7, cellular uptake experiments were conducted in the presence of human epidermal growth factor (EGF, Sigma-Aldrich) (100 nM).

In the macropinocytosis inhibition assay, the cells were pretreated with the macropinocytosis inhibitor 5-(N-ethyl-N-isopropyl) amiloride^16^,^21^ (EIPA, Sigma-Aldrich) in MEM containing 10% FBS (100 μM, 600 μl/well) for 30 min at 37 °C prior to treatment with each sample (600 μl/well) and in MEM containing 10% FBS (200 μl/well) in the presence or absence of EIPA (100 μM) for 1 h at 37 °C prior to flow cytometry analysis.

### Electron microscopy

Suspended EVs in PBS (30 μg/ml) were dropped onto a carbon-coated grid (400 mesh) and washed with distilled water. Uranyl acetate was applied to the grid and left for 10 s at room temperature. Next, the reagent was removed with filter paper and dried prior to imaging with a transmission electron microscope (TEM) (JEM1200EX, JEOL, Tokyo, Japan).

### Zeta-potential and particle size

The zeta-potential and particle size of the EVs diluted in PBS (58.2 μg/ml) were determined using the zeta-potential and particle size analyzer ELSZ-DN2 (Otsuka Electronics, Osaka, Japan) according to the manufacturer’s instructions.

### Cell ruffling assay

HeLa cells (2 × 10^5^ cells, 2 ml) were plated onto a 35-mm glass dish (Iwaki) and incubated in cell culture medium (α-MEM) containing 10% FBS for 24 h at 37 °C under 5% CO_2_. After complete adhesion, the cells were washed with cell culture medium containing 10% FBS and treated with each EV sample (100 μl/well) for 20 min at 37 °C. After removal of the medium, the cells were fixed with 4% paraformaldehyde at room temperature for 30 min and washed with PBS. The cells were then treated with 0.1% Triton X-100 (100 μl/well in PBS) at room temperature for 5 min and again washed with PBS. Cellular F-actin was stained with rhodamine-phalloidin (Molecular Probes) for 20 min at 4 °C, and the cells were washed with PBS prior to analysis using a FV1200 confocal laser scanning microscope (Olympus) equipped with a 40× objective.

### Preparation of FITC-labeled saporin

For the preparation of FITC-labeled saporin, saporin (200 μg, saporin from *Saponaria officinalis* seeds, Sigma-Aldrich) dissolved in H_2_O (100 μl) was reacted with 2 equivalents of FITC (Sigma-Aldrich) dissolved in dimethyl sulfoxide (10 μl) and N,N-diisopropylethylamine (0.5 μl) at 30 °C for 2 h as previously reported^9^,^10^. To remove the unreacted FITC, gel filtration on a Sephadex G-25 column (PD-10, GE Healthcare) was performed prior to lyophilization. The protein concentration was determined using a Pierce BCA protein assay kit.

### Encapsulation of fluorescently labeled dextran and saporin into EVs

To load the fluorescently labeled dextran into EVs, EVs (25 μg) were mixed with FITC-labeled dextran (molecular weight: 70,000) (Sigma-Aldrich) or saporin (50 μg) in PBS (100 μl). After electroporation (poring pulse: two pulses (200 V, 5 msec); transfer pulse: five pulses (20 V, 50 msec)) in a 1-cm electroporation cuvette at room temperature using a super electroporater NEPA21 Type II (NEPA Genes, Tokyo, Japan), the unencapsulated FITC-dextran or saporin was removed by washing and filtration using Amicon Ultra centrifugal filters (100 K device), as previously reported^9^,^10^. Loading of FITC-dextran and FITC-saporin into EVs was confirmed using a spectrofluorometer (FP-6200, JASCO, Tokyo, Japan)^9^,^10^, and the loading concentration was calculated on the basis of the calibration curve of FITC (excitation: 488 nm; emission: 530 nm). The electroporation method resulted in the encapsulation of FITC-dextran (5 ng/ml) in 1 μg/ml of EVs. The efficiency of dextran encapsulation into EVs was calculated to be 0.3%. The concentration of saporin encapsulated in 10 μg/ml EVs was estimated to be approximately 43.5 ng/ml using the FITC-labeled saporin. The efficiency of saporin encapsulation into EVs was calculated to be 0.2%.

### Cell viability (WST-1 assay)

Cell viability was analyzed using the WST-1 (4-[3-(4-iodophenyl)-2-(4-nitrophenyl)-2H-5-tetrazolio]-1,3-benzene disulfonate) assay, as previously described^9^,^10^. Cells (1 × 10^4^ cells, 100 μl) were incubated in 96-well microplates in cell culture medium containing 10% FBS for 24 h at 37 °C under 5% CO_2_. The cells were then treated with each EV sample (50 μl) at 37 °C under 5% CO_2_. After the sample treatment, WST-1 reagents (10 μl) were added to each well and the samples were incubated for 40 min at 37 °C. The absorbencies at 450 nm (A450) and 620 nm (A620) were measured, and the value obtained by subtracting A620 from A450 corresponded to the viable cell number.

#### Statistical analyses

All statistical analyses were performed using GraphPad Prism software (ver. 5.00; GraphPad, San Diego, CA, USA). For comparisons of two groups, unpaired Student’s *t*-test was used after verification of the equal variances with an F-test. Welch’s correction was performed when the variances across groups were assumed to be unequal. For multiple comparison analyses, one-way analysis of variance (ANOVA) followed by Tukey’s post hoc test or Dunnett’s post hoc test was used. Differences were considered significant when the calculated *p*-value was < 0.05.

## Additional Information

**How to cite this article**: Nakase, I. *et al.* Vectorization of biomacromolecules into cells using extracellular vesicles with enhanced internalization induced by macropinocytosis. *Sci. Rep.* **6**, 34937; doi: 10.1038/srep34937 (2016).

## Supplementary Material

Supplementary Information
