# Supplementary material for: Vectorization of biomacromolecules into cells using extracellular vesicles with enhanced internalization induced by macropinocytosis
Source: Sci Rep. 2016 Oct 17;6:34937. doi: 10.1038/srep34937 (PMC5066177; doi:10.1038/srep34937)
Supplement: Supplementary Information [file srep34937-s1.pdf]

Scientific Reports

Supplementary information

Title:

Vectorization of biomacromolecules into cells using extracellular vesicles with enhanced internalization induced by macropinocytosis

Authors:

Ikuhiko Nakase[1,*], Kosuke Noguchi[1,2], Ikuo Fujii[2], Shiroh Futaki[3]

Affiliations:

[1]Nanoscience and Nanotechnology Research Center, Research Organization for the 21st Century, Osaka Prefecture University, Naka-ku, Sakai, Osaka 599-8570, Japan

[2]Graduate School of Science, Osaka Prefecture University, Naka-ku, Sakai, Osaka 599-8531, Japan

[3]Institute for Chemical Research, Kyoto University, Uji, Kyoto 611-0011, Japan

*Correspondence and requests for materials should be addressed to I.N. (email: i-nakase@21c.osakafu-u.ac.jp).

Supplementary Figures

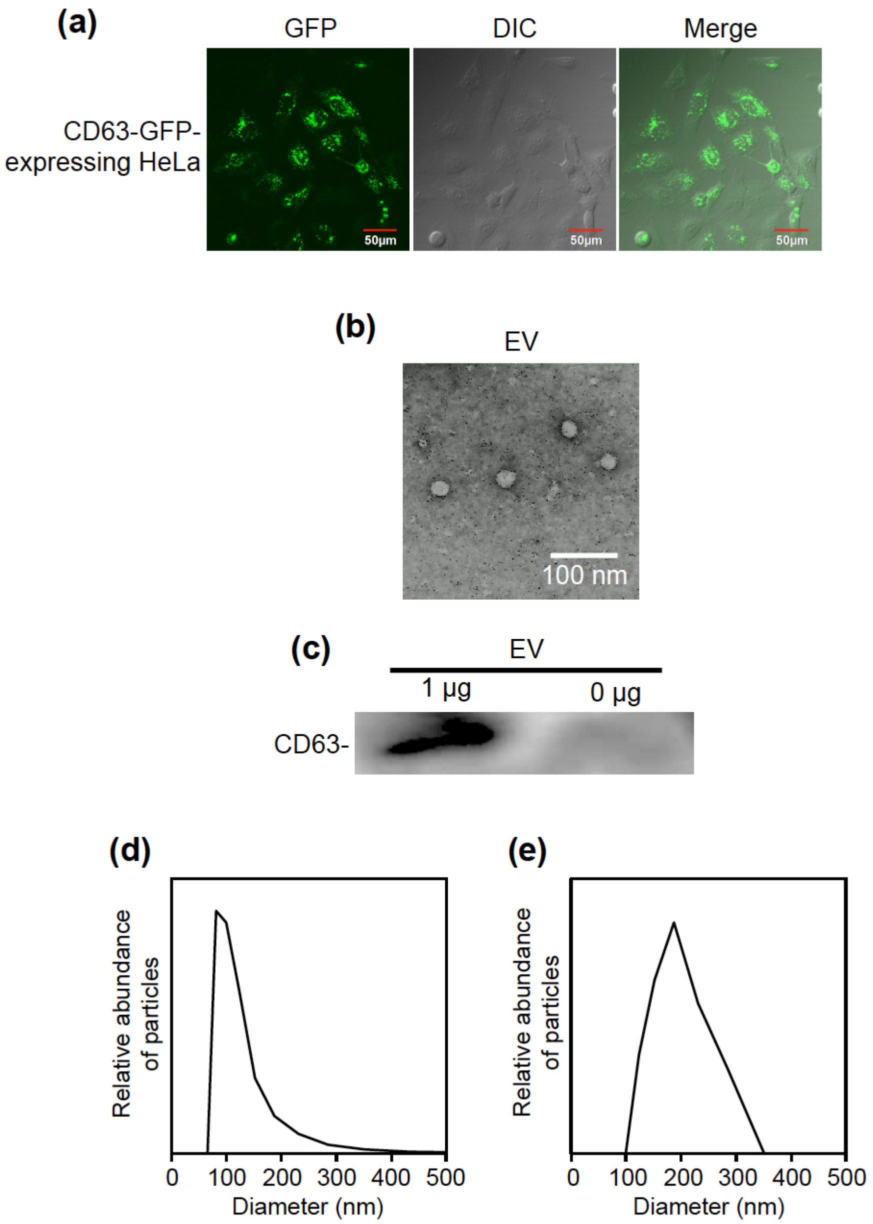

Supplementary Figure 1. **Secretion of CD63-GFP EVs from HeLa cells stably expressing CD63-GFP.** (**a**) Confocal microscopic images of HeLa cells stably expressing CD63-GFP. (**b**) TEM observation of isolated CD63-GFP-EVs. (**c**) Western blot analyses showing EVs secreted from HeLa cells. The CD63 EV marker protein was detected as described in the Methods section. Detection of immunoreactive species using anti-CD63 was the position at approximately 50 kDa using SDS-PAGE analysis. (**d, e**) Size distributions of CD63-GFP-EVs (**d**) and stearyl-r8-modified CD63-GFP-EVs (**e**) analyzed using a particle size analyzer.

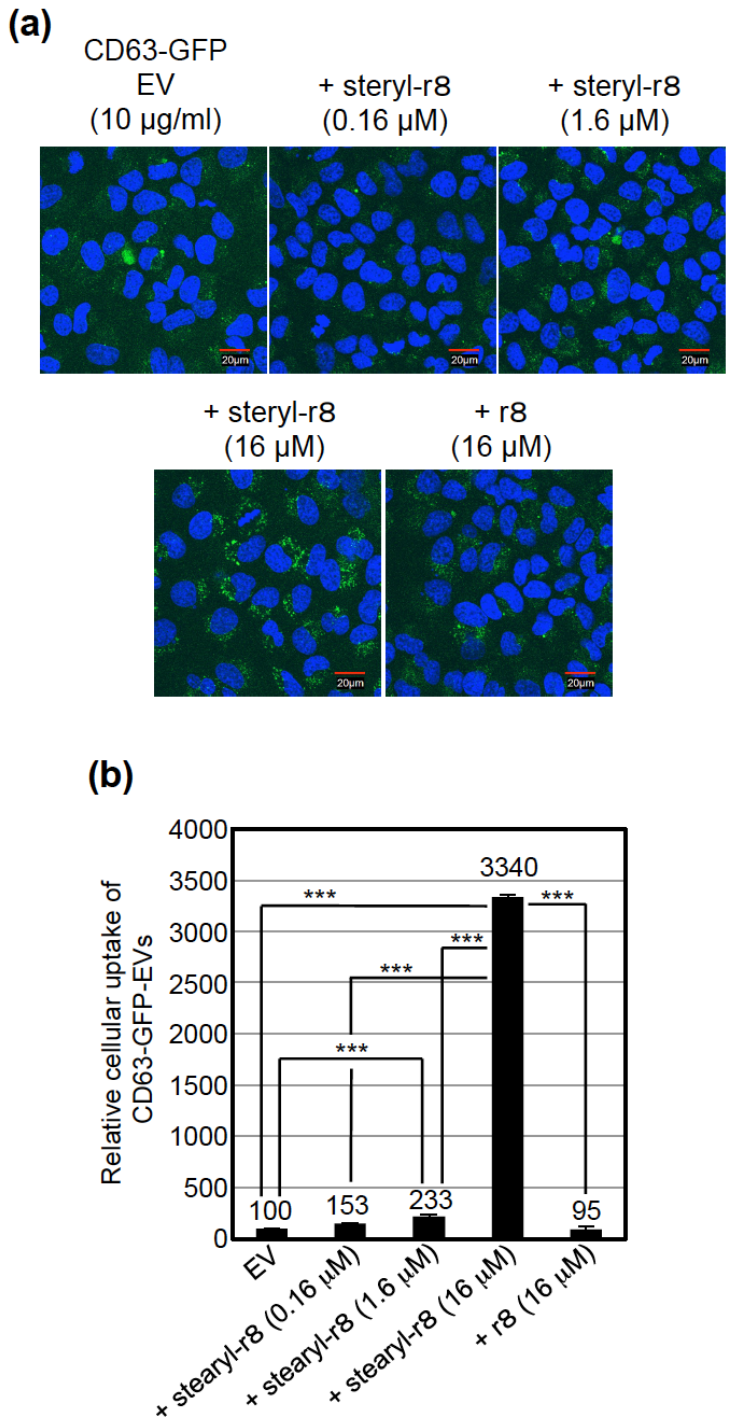

Supplementary Figure 2. **Optimization of stearyl-r8 peptide modification on EV membranes for the effective cellular uptake of EVs.** (**a**) Confocal microscopic image of HeLa cells treated with CD63-GFP-EVs (10 μg/ml) modified with stearyl-r8 (0.16-16 μM) or r8 without a stearyl moiety (16 μM) for 24 h at 37°C (blue: Hoechst 33342; green: CD63-GFP

EVs). (**b**) Relative cellular uptake of CD63-GFP-EVs (10 μg/ml) modified with stearyl-r8 (0.16-16 μM) or r8 (16 μM) analyzed using a flow cytometer under the same experimental conditions as (**a**). The data are expressed as the average (±SD) of three experiments. $^{***}p <$ 0.001.

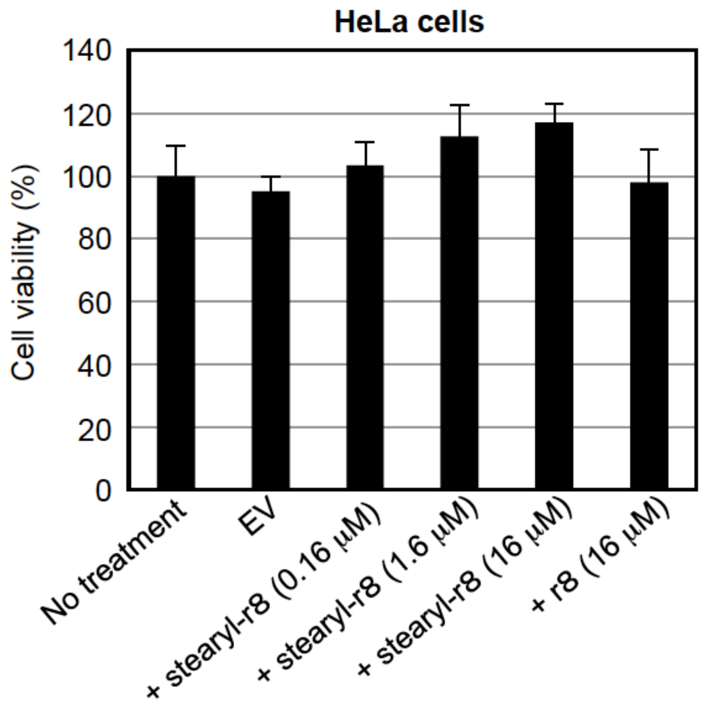

Supplementary Figure 3. **Cell viability.** HeLa cells were treated with EVs (10 μg/ml) modified with stearyl-r8 (0.16-16 μM) or r8 (16 μM) for 24 h at 37°C prior to WST-1 assay. The data are expressed as the average (±SD) of four experiments.

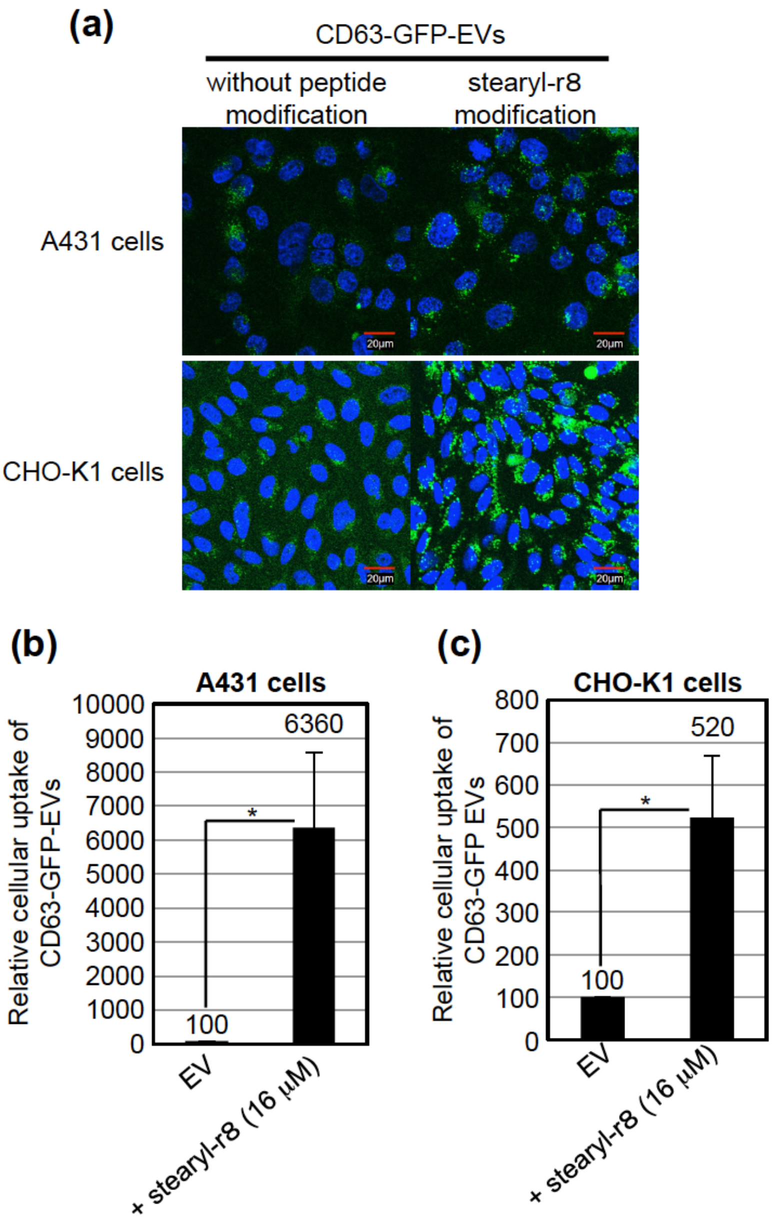

Supplementary Figure 4. **Stearyl-r8 peptide modification on the EV membrane enhances cellular EV uptake.** (**a**) Confocal microscopic image of A431 or CHO-K1 cells treated with CD63-GFP-EVs (10 μg/ml) modified with or without stearyl-r8 (16 μM) for 24 h at 37°C (blue: Hoechst 33342; green: CD63-GFP-EVs). (**b**, **c**) Relative cellular uptake (A431 (**b**) or CHO-K1 (**c**)) of CD63-GFP-EVs (10 μg/ml) modified with or without stearyl-r8 (16 μM) analyzed using a flow cytometer under the same experimental conditions as (**a**). The data are expressed as the average (±SD) of three experiments. $^{*}p < 0.05$.

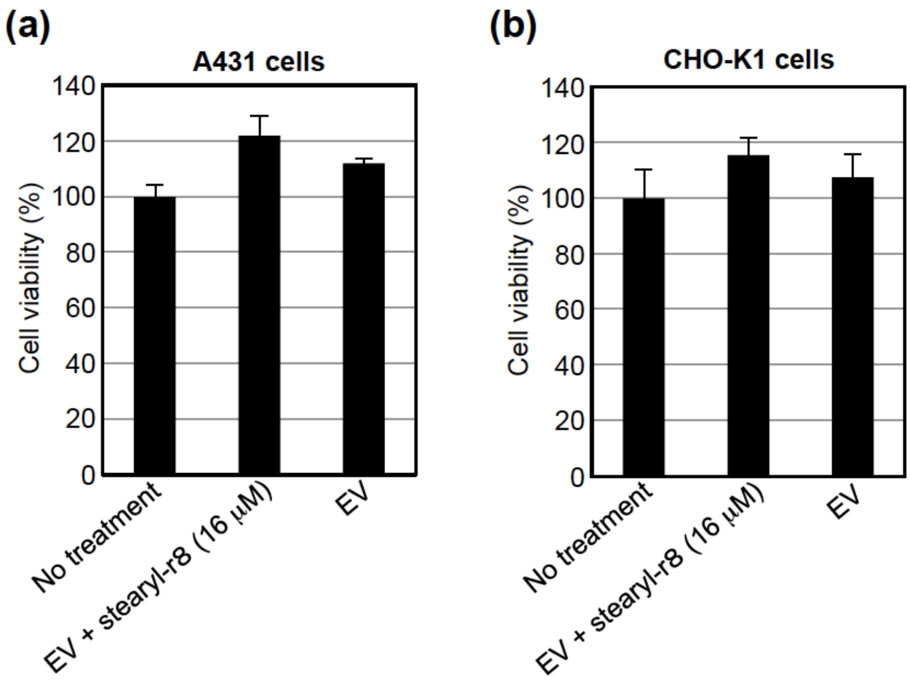

Supplementary Figure 5. **Cell viability.** (**a**, **b**) A431 (**a**) or CHO-K1 (**b**) cells were treated with EVs (10 μg/ml) modified with or without stearyl-r8 (16 μM) for 24 h at 37°C prior to WST-1 assay. The data are expressed as the average (±SD) of four experiments.

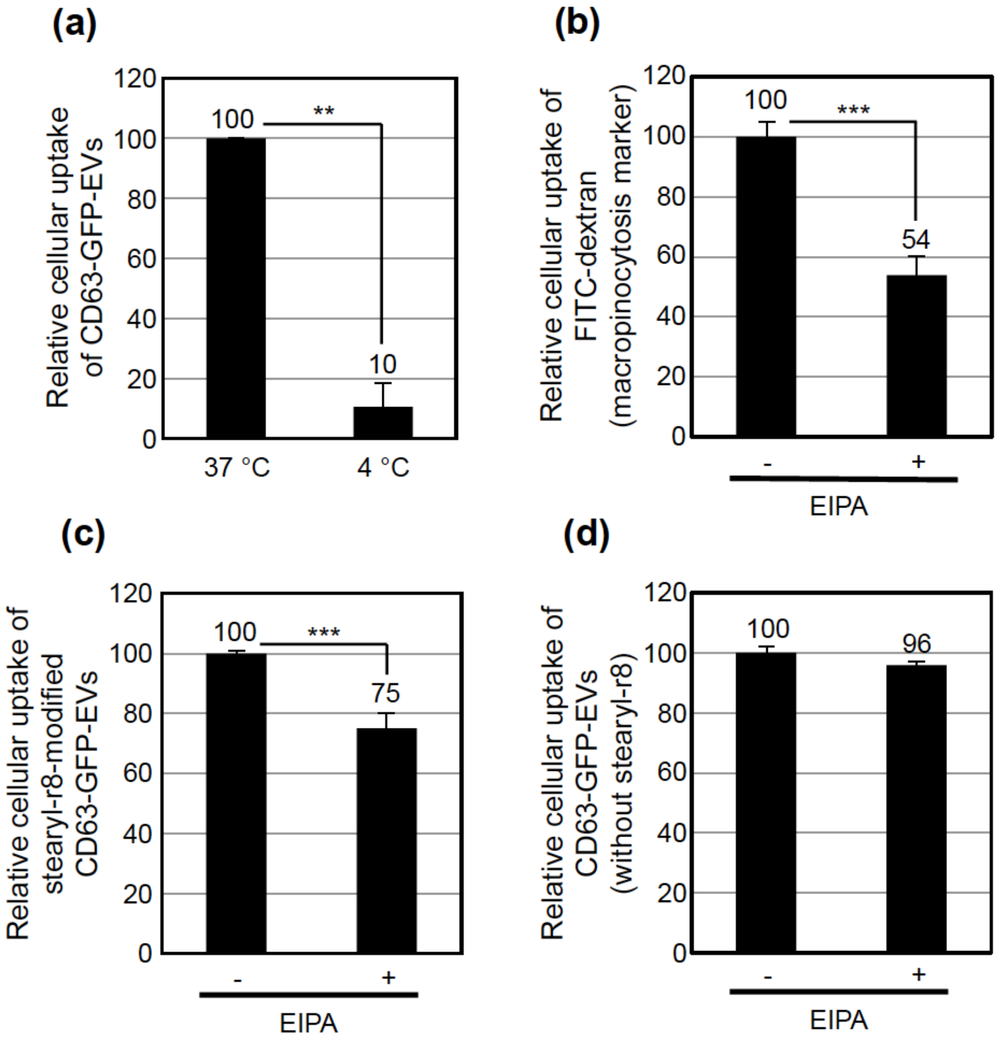

Supplementary Figure 6. **Flow cytometry analysis under experimental conditions of endocytosis or macropinocytosis prevention.** (**a**) Relative cellular uptake (HeLa cells) of CD63-GFP-EVs (10 μg/ml) modified with stearyl-r8 (16 μM) for 2 h at 37°C or 4°C for the prevention of endocytosis analyzed using a flow cytometer. (**b-d**) Relative cellular uptake (HeLa cells) of the macropinocytosis marker FITC-dextran (molecular weight: 70,000) (**b**), CD63-GFP EVs (10 μg/ml) modified with stearyl-r8 (16 μM) (**c**), and CD63-GFP-EVs (10 μg/ml) without the modification of stearyl-r8 (**d**) for 1 h at 37°C in the presence or absence of the macropinocytosis inhibitor EIPA (100 μM) analyzed using a flow cytometer. In the experimental condition of (**b**), the cells were also treated with epidermal growth factor (EGF, 100 nM) for the induction of macropinocytosis via the activation of the epidermal growth factor receptor. The data are expressed as the average (±SD) of three experiments. $^{**}p < 0.01$, $^{***}p < 0.001$.

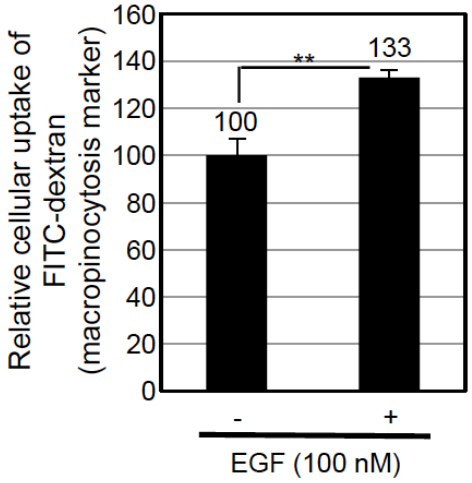

Supplementary Figure 7. **Activation of epidermal growth factor receptor enhances macropinocytotic cellular uptake.** Relative cellular uptake (HeLa cells) of the macropinocytosis marker FITC-dextran (molecular weight: 70,000) for 24 h at 37°C in the presence or absence of EGF (100 nM) analyzed using a flow cytometer. The data are expressed as the average (±SD) of three experiments. $^{**}p < 0.01$.

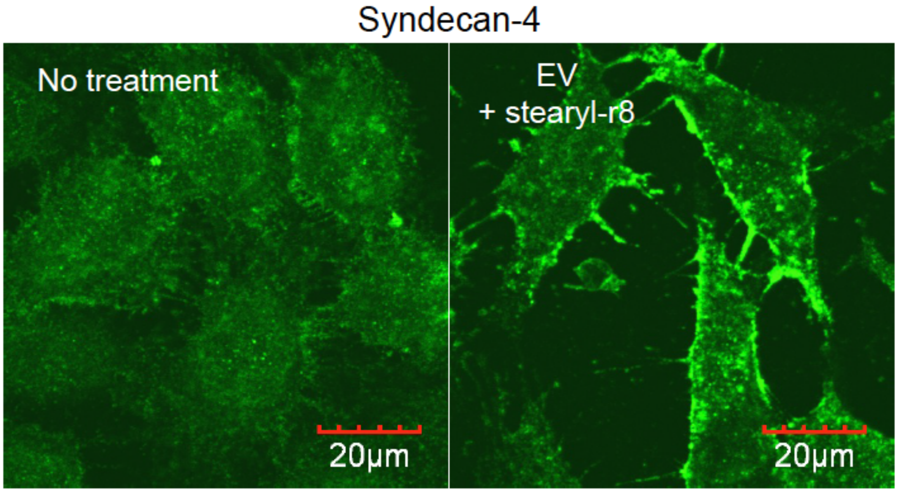

Supplementary Figure 8. **Enhanced clustering of syndecan-4 on the plasma membrane via the treatment of stearyl-r8-modified EVs.** Confocal microscopic image of HeLa cells treated with EVs (without CD63-GFP expression, 10 μg/ml) modified with stearyl-r8 (16 μM) for 1 h at 37°C. Antibody staining for syndecan-4 (green: syndecan-4 antibody) was performed prior to the observation.

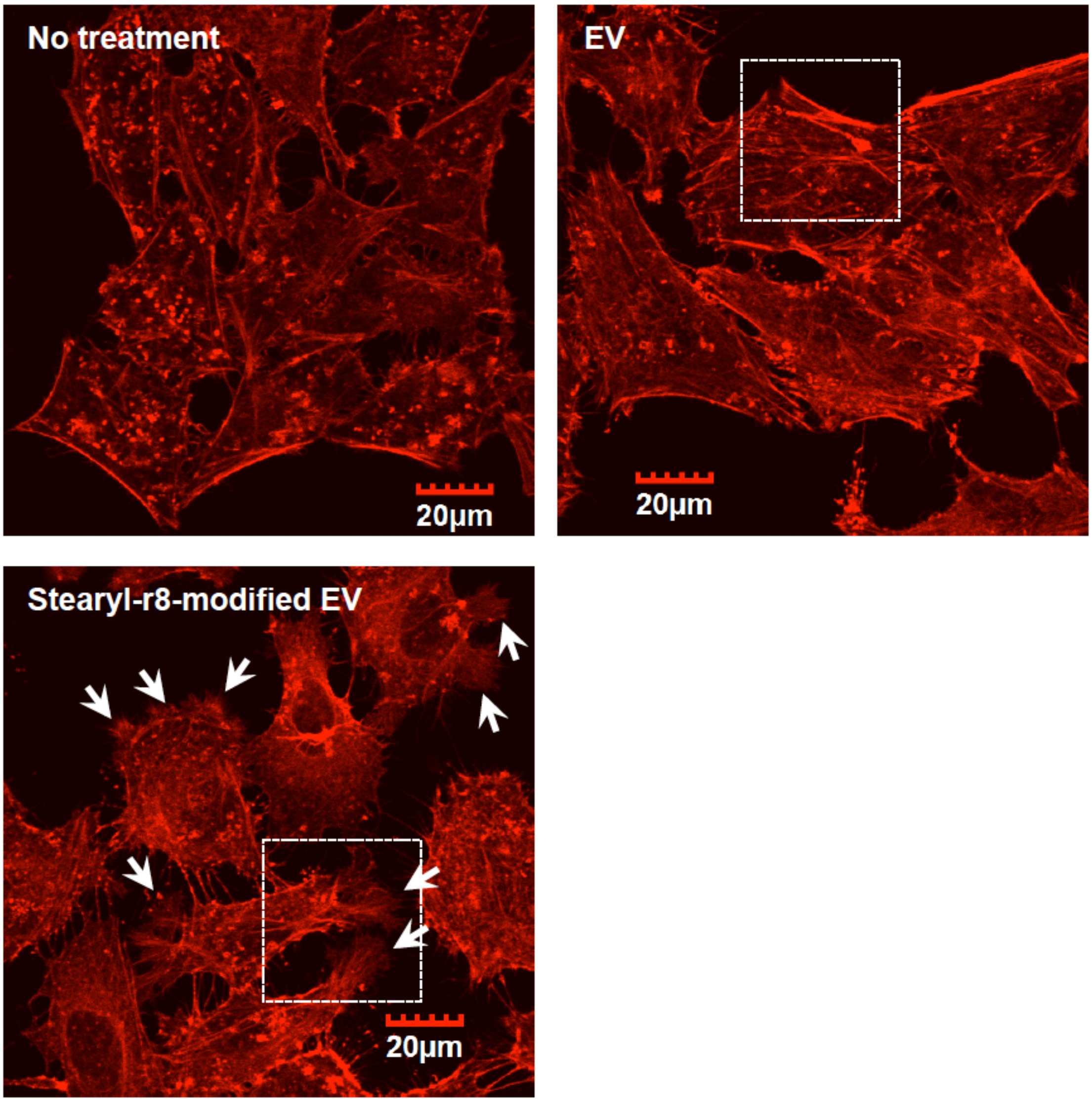

Supplementary Figure 9. **Induction of lamellipodia formation by stearyl-r8-modified EVs.** Confocal microscopic observation of HeLa cells treated with EVs (10 μg/ml) modified with stearyl-r8 (16 μM) or EVs without the peptide modification for 20 min at 37°C. Cellular staining with rhodamine-phalloidin was performed to visualize F-actin prior to the observation. Enlarged images of the areas marked by white squares are shown in Figure 3c. The arrows indicate representative lamellipodia formations.

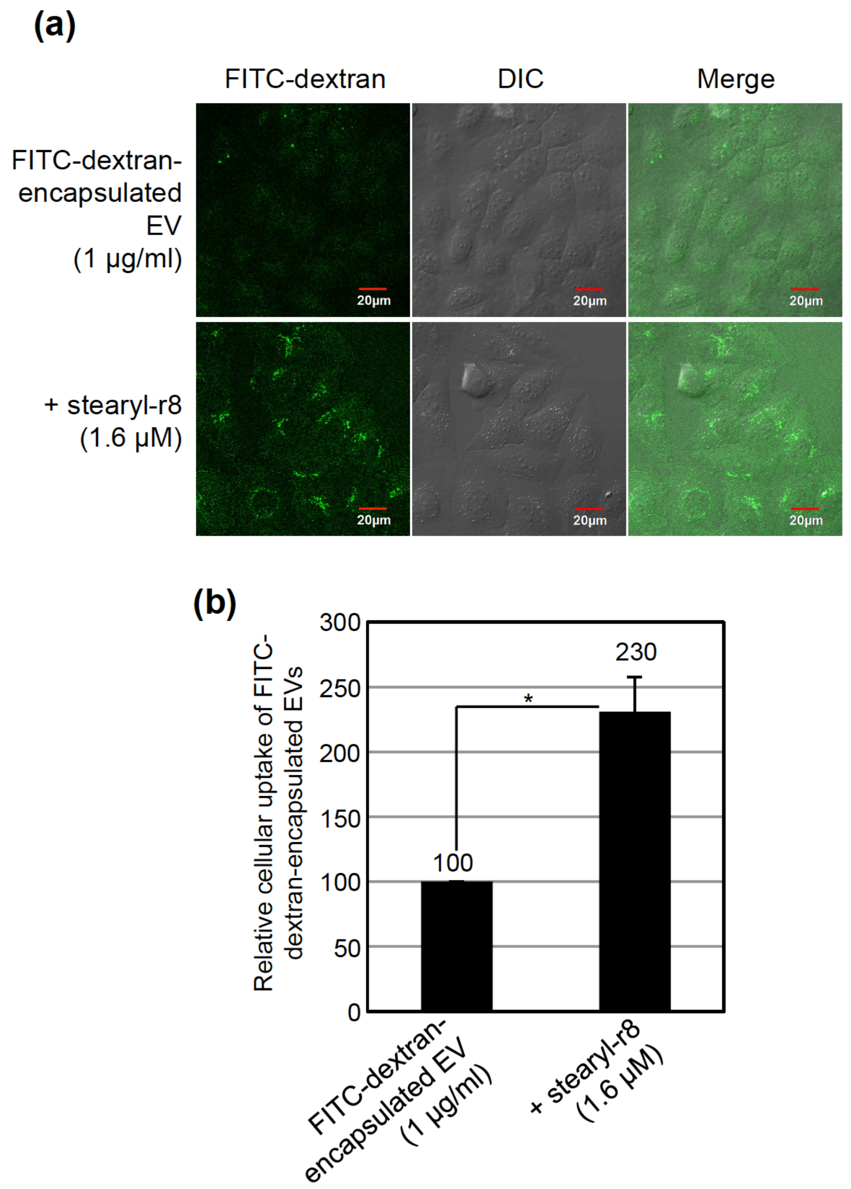

Supplementary Figure 10. **The cellular uptake efficacy of FITC-dextran-encapsulated EVs is enhanced by the modification of stearyl-r8 on the EV membrane.** (**a**) Confocal microscopic image of HeLa cells treated with FITC-dextran-encapsulated EVs (without CD63-GFP expression, 1 μg/ml) modified with or without stearyl-r8 (1.6 μM) for 24 h at 37°C (green: FITC-dextran-encapsulated EVs). (**b**) Relative cellular uptake of FITC-dextran-encapsulated EVs (without CD63-GFP expression, 1 μg/ml) modified with or without stearyl-r8 (1.6 μM) analyzed using a flow cytometer under the same experimental conditions as in (**a**). The data are expressed as the average (±SD) of three experiments. $^{*}p < 0.05$.
